# Hydrogen Analysis by Gas Chromatography–Mass Spectrometry

**DOI:** 10.1021/acs.analchem.6c00257

**Published:** 2026-05-22

**Authors:** Vladislav V. Lobodin, Yensil Park, Charles E. A. Finney

**Affiliations:** Buildings and Transportation Science Division, 6146 Oak Ridge National Laboratory, Oak Ridge, Tennessee 37831-6472, United States

## Abstract

The detection of hydrogen in complex gas mixtures is essential for many applications. Conventional approaches such as gas chromatography (GC) with thermal conductivity detection (TCD) and residual gas analyzers (RGAs) face significant limitations: TCD exhibits poor response when helium is used as a carrier gas, whereas RGAs lack chromatographic separation, preventing reliable quantification of hydrogen because of interference from other species. Traditional GC methods rely on dual-column configurations with packed or molecular-sieve porous layer open tubular (PLOT) columns to separate hydrogen from O_2_, N_2_, CO, CO_2_, CH_4_, and other hydrocarbons, increasing system complexity and limiting compatibility with mass spectrometry (MS) detection. In this work, we developed and validated a robust GC–MS method capable of directly detecting and quantifying hydrogen using electron ionization (EI) without dopants, reagent gases, or ion–molecule reaction schemes. By integrating a modified EI source and a cryogenically cooled single capillary column configuration, we achieved baseline separation of hydrogen from all major permanent gases and hydrocarbons in a refinery gas mixture using helium as the carrier gas. The method demonstrated excellent linearity, high sensitivity, and exceptional reproducibility. Adjustable sample-loop volumes and split ratios enabled optimization of peak shape and signal-to-noise performance for trace-level and percent-level hydrogen concentrations. Beyond hydrogen quantitation, the method provides simultaneous compositional profiling of other gases in a mixture in a single run, making it valuable for a wide range of tasks.

## Graphical abstract

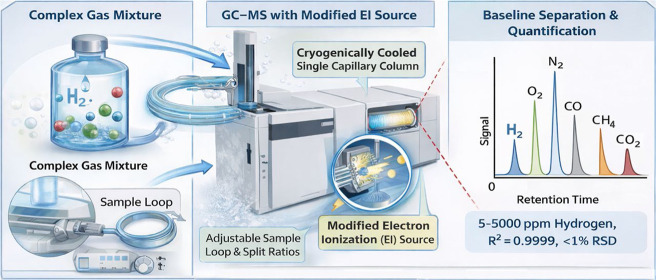

## Introduction

Hydrogen analysis in complex gas mixtures, including permanent gases, and volatile and semivolatile organic and inorganic compounds, is crucial for optimizing hydrogen production, storage and use, and ensuring reliability across diverse applications. Accurate hydrogen analysis underpins production and distribution processes such as steam methane reforming, gasification, and electrolysis, helping maintain purity and quality throughout the supply chain.^1^ Reliable hydrogen measurements are also critical for fuel cell power generation, where automotive manufacturers and fueling stations must meet strict fuel quality standards to ensure safety and performance.^2,3^ Hydrogen is a flammable, colorless, and odorless gas that poses significant hazards in air at levels between 4 and 74%.^4,5^

Many industrial processes, including chemicals and petrochemicals, steel manufacturing, glass production, and electronics, depend on precise hydrogen measurements for process control, quality assurance, and energy efficiency.^6–9^ Beyond these established uses, hydrogen analysis plays a vital role in energy storage, power generation, and utilization as a fuel for transportation and grid applications (e.g., power-to-gas and sustainable integration), with monitoring required to maintain the integrity and safety of stored and injected hydrogen.^10,11^ It is also essential for environmental monitoring, including leak detection, emissions control,^5,12^ and geological exploration of natural hydrogen sources.^13–15^ In the aerospace industry, hydrogen analysis ensures its safe handling and optimal performance of hydrogen as a fuel or propellant.^16–18^ In addition, forensic studies also rely on hydrogen measurements to provide critical evidence in criminal investigations and legal cases.^19,20^

A GC coupled to thermal conductivity detector (TCD) continues to be a common method of choice for hydrogen analysis despite the detector limitations.^21,22^ However, the use of helium as a carrier gas, despite being the most popular choice, results in complications with a TCD because the thermal conductivity for hydrogen and helium is very close and hydrogen produces a relatively low and nonlinear response. To avoid the hydrogen–helium interference, argon or nitrogen is used as the carrier gas with a TCD. However, the use of those gases compromises chromatographic resolution, significantly reduces sensitivity for the other compounds by a factor of ∼10, and can interfere with the detection of other analytes.

Besides a TCD, a helium ionization detector (HID) and an atomic emission detector (AED) are utilized by coupling to a GC to analyze hydrogen.^23,24^ As in the case of a TCD, these detectors suffer from limitations and more importantly are not informative enough for identification of unknown chromatographic peaks during the hydrogen production development. Raman spectroscopy can detect low hydrogen concentrations but suffers from low sensitivity, complex setups, and long measurement times. Commercial systems have high detection limits and reduced precision over time, limiting practicality for dynamic or in-line applications.^25,26^

In contrast, GC coupled to MS serves as a powerful tool for both qualitative and quantitative analysis of various compound mixtures.^27^ Nonetheless, it has been reported that GC–MS faces challenges due to the difficulties of detecting lighter gases relative to the carrier gas^28,29^ and direct EI in the absence of a dopant and ion–molecule reactions has been considered an impossible task.^29^

Chromatographic analysis of hydrogen typically involved a dual-column setup to effectively separate it from other gases like O_2_, N_2_, CO, CO_2_, CH_4_, and various volatile hydrocarbons. These setups often used packed columns or molecular sieves PLOT columns, which additionally necessitated a particle trap on the detector side to block sorbent particles from entering the mass spectrometer’s ion source.^30,31^

In the present study, we report the development of a novel method for hydrogen analysis that employs direct EI of hydrogen and a simplified column configuration with a single capillary column. This approach is capable of detecting hydrogen across a broad concentration range, including trace levels in complex matrices. Importantly, our method also delivers compositional information for other gases in a single run, which can provide valuable context for many applications, thereby providing a significant advancement in hydrogen analysis methodology.

## Experimental Part

The development of the method for hydrogen analysis was conducted with a GC–MS instrument employing an Agilent 7890B GC system and an Agilent 5977B mass spectrometer (Agilent Technologies, Santa Clara, CA). The GC system was modified with a liquid N_2_ cryogenic valve kit (Agilent G3466A) to enable operation at low temperatures and liquid N_2_ (supplied from a Dewar) was used to cool the GC oven. Chromatography-grade helium with a purity of 99.9999% from Airgas (Radnor, PA) additionally passed through a helium purifier trap (model RMSH-2) from Agilent Technologies at a flow rate of 2.0 mL·min^–1^ was used as a carrier gas. The gas chromatograph inlet temperature was set to 200 °C. The GC oven temperature was programmed to hold at −80 °C for 2 min, subsequently ramped to 260 °C at 20 °C·min^–1^, and then held for 17 min. The transfer line was kept at 260 °C. A GS-GASPRO GC column (60 m long, 320 μm ID) from Agilent Technologies was used for chromatographic separation. The ion source was held at 230 °C. The EI ion source was modified by replacing a standard (350 G) magnet with a low-gauss magnet (220 G) assembly (Agilent part: G3163-60560). The hydrogen tune macros were procured from Diablo Analytical (Antioch, CA) and are described elsewhere.^32^ Both selected ion monitoring (SIM) and scan modes (recorded as a total ion chromatogram [TIC]) were utilized for detection of hydrogen and other gases. In particular, hydrogen detection was performed using SIM mode at a mass-to-charge ratio (*m*/*z*) of 2.

A valve box kit (Agilent G1580A) with a six-port valve was installed for gas sample introduction. Various volumes of sample loops (0.250, 0.500, 1, and 2 mL) were supplied from Vici Valco Instruments (Houston, TX). Certified standard gas mixtures of helium in concentrations of 50000 (5%), 5000, 500, 50, and 5 ppm with nitrogen as a balance gas (procured from Airgas) were used to optimize hydrogen detection. To optimize chromatographic performance, we used a refinery gas test mixture (purchased from Agilent Technologies) containing gases in the following concentration (volume %): 15% hydrogen, 5% propane, 1% propylene, 10% iso-butane, 2% iso-pentane, 1% *n*-pentane, 15% nitrogen, 5% methane, 5% *n*-butane, 10% 1-butene, 5% *trans*-2-butene, 5% *cis*-2-butene, 5% carbon dioxide, 5% carbon monoxide, 1% ethylene, 10% ethane, and nitrogen as a balance gas (remaining volume). The hydrogen quantitation results are presented as average values from triplicate analyses ± the standard deviation.

## Results and Discussion

Indirect methods of hydrogen detection using chemical ionization (CI) and EI ion sources in GC–MS involving ion–molecular reactions have been previously reported.^29–31,33,34^ EI without the use of a dopant or ion–molecule reactions has traditionally been impractical for hydrogen detection because H_2_ has a high ionization energy (15.43 eV) and an extremely low ionization cross-section. A standard EI source in GC–MS instruments is optimized for higher mass analytes and inherently discriminates against low-mass ions (*m*/*z* < 10), such as H_2_^+^ (*m*/*z* = 2). Additionally, hydrogen’s low mass makes it highly susceptible to interference from residual gases, vacuum background, and detector limitations. Unlike heavier compounds, hydrogen lacks fragmentation pathways that could provide alternative diagnostic ions, further limiting detectability. Consequently, prior approaches have relied on dopants or ion–molecule reactions to generate more detectable species. However, these methods introduced other operational challenges and interferences that can affect the reliability of hydrogen analysis.

Here, we describe the development of a new hydrogen analysis approach that utilizes direct EI and selective detection at *m*/*z* = 2. Because the conventional EI source magnetic field is optimized to confine and focus electrons for ionization of species with *m*/*z* ≥ 10, this configuration is suboptimal for hydrogen, resulting in limited ionization efficiency and inefficient extraction of low-mass ions. The modification of the EI ion source with a low-gauss magnet is necessary to improve the formation and detection of H_2_^+^ ions. Reduction of the magnetic field strength decreases confinement of ionizing electrons emitted by the filament, thereby increasing the effective interaction volume and residence time of electrons with hydrogen molecules and enhancing ionization probability. In addition, the reduced magnetic field mitigates discrimination against low-mass ions during extraction and transmission into the mass analyzer. Collectively, these effects enhance sensitivity for H_2_ detection under EI conditions, eliminating the need for chemical ionization or indirect detection strategies.

In addition to the specialized hardware, custom low-mass optimization tune macros are required to achieve reliable sensitivity. We used tune macros from a hydrogen detection kit developed for Agilent MSD instruments that had been converted into standalone RGA-type mass analyzers.^32^ The tuning and mass-calibration procedure began with a low-mass autotune (lomass.u), followed by a helium optimization tune that used the helium carrier gas flowing through the column into the ion source as the tuning mixture. The optimization tune adjusts key ion–optics parameters, including repeller voltage, ion focus, electron energy, emission current, and entrance lens offset to maximize the response at *m*/*z* = 4. Additionally, ions at *m*/*z* = 69, 131, and 219 from the instrument’s calibration compound (PFTBA) were used to calibrate the mass axis, adjust the electron multiplier voltage, and set appropriate peak widths.

The mass spectrometry detection method involved simultaneous acquisition in SIM mode at *m*/*z* = 2 for hydrogen detection, and scan mode (at 2.4 scans s^–1^) within the range of *m*/*z* = 10–160 for other gases. Attempts to scan from *m*/*z* = 1.6 to 160 and subsequently extract the ion chromatogram at *m*/*z* = 2 resulted in a significantly lower signal response for hydrogen detection. Detection of hydrogen in SIM mode at *m*/*z* = 2 significantly improves the signal-to-noise (S/N) ratio and detection limit. The dwell time (the time during which the quadrupole is set at *m*/*z* = 2) was adjusted to 100 ms to enhance sensitivity. The high-resolution quadrupole settings (with peak width of 0.5 *m*/*z*) further improved S/N ratio by a factor of 2.

Standalone mass spectrometers (without GC) are often employed as RGAs, including for hydrogen detection. However, the absence of chromatographic separation limits reliable hydrogen quantification and comprehensive characterization of other species in a mixture. Furthermore, quadrupole mass spectrometers, commonly used both as RGAs and in GC–MS configurations, are subject to the “zero-blast” effect.^35^ This occurs when low DC voltage and RF amplitudes at low *m*/*z* are insufficient to prevent heavier ions from reaching the detector, leading to false signals in the low *m*/*z* range up to *m*/*z* = 4, even in the absence of corresponding low molecular weight analytes such as H_2_ or He.^36^ The “zero-blast” effect is particularly noticeable when measuring low levels of hydrogen amidst significantly higher concentrations of heavier gases, e.g., N_2_, O_2_, CO, Ar, CO_2_, H_2_O, hydrocarbons. This interference, which fluctuates constantly, makes the detection and resolution of hydrogen peaks at *m*/*z* = 2 highly challenging. Figure 1 shows the GC–MS analysis of two standard gas mixtures containing 50000 and 5000 ppm hydrogen with nitrogen as the balance gas. The injected sample volume was 250 μL, and the split ratio was 20:1. The upper plots display total ion chromatograms for the mass range *m*/*z* = 10–160, where the peak at 4.1 min corresponds to N_2_. The lower plots show extracted ion chromatograms at *m*/*z* = 2, where the peak at 3.4 min represents H_2_, while the peak at 4.1 min, matching the nitrogen elution time, is a false signal caused by the “zero-blast” effect. This interference is especially pronounced at lower hydrogen concentrations and would significantly affect quantitation in the absence of chromatographic resolution. By separating hydrogen into a distinct chromatographic peak with GC–MS, the “zero-blast” interference can be minimized. Therefore, efficient chromatographic separation of hydrogen from other gases is essential for reliable detection and quantification.

**Figure 1:**
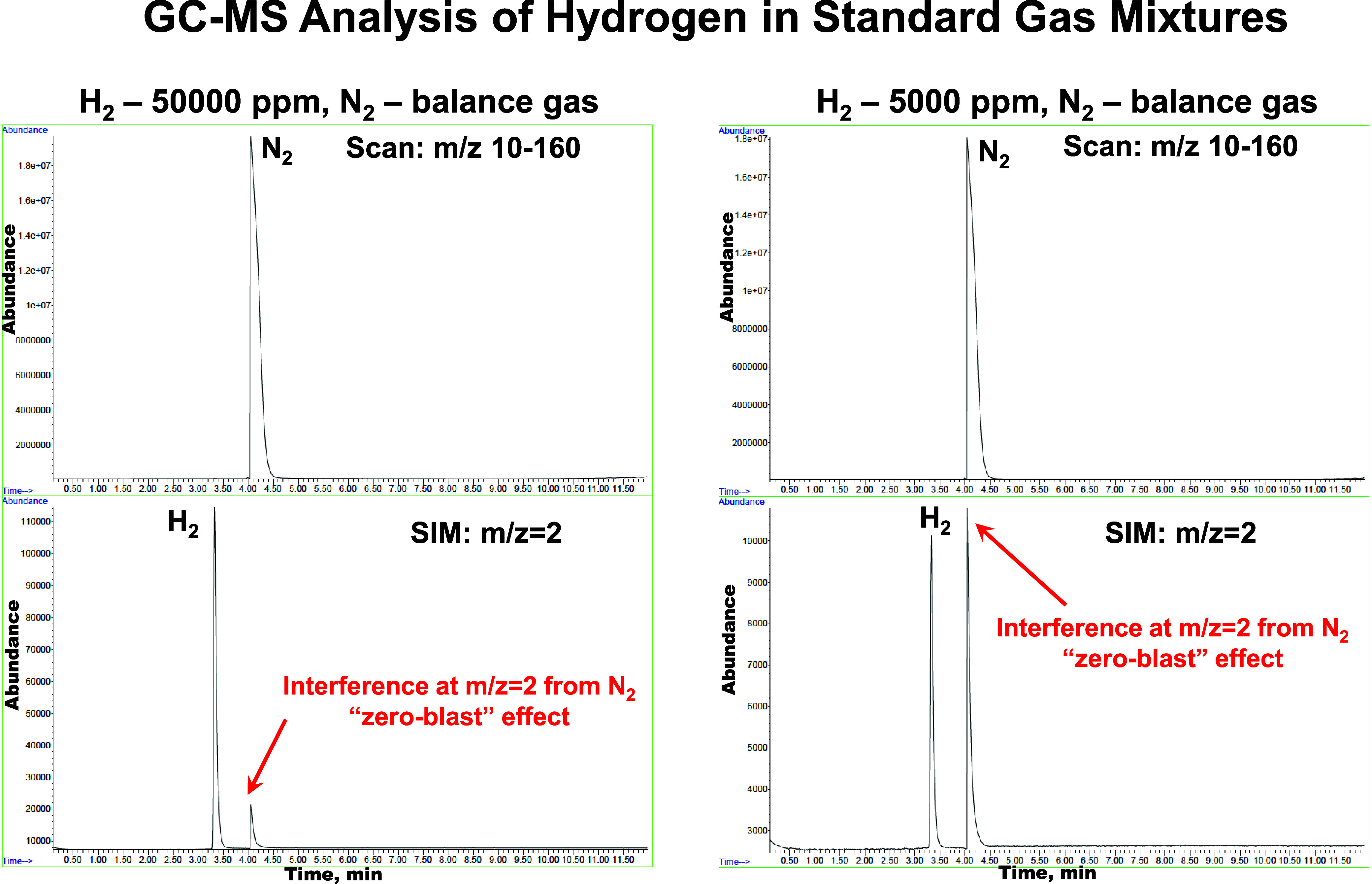
GC–MS analysis of hydrogen standard gas mixtures. Upper figures show scans from *m*/*z* = 10–160, and lower figures show detection at *m*/*z* = 2. Left: 5% (50000 ppm) H_2_. Right: 5000 ppm of H_2_. Nitrogen is a balance gas.

To simplify chromatographic analysis of hydrogen - which typically requires a two-column configuration and specific settings (e.g., a wider-ID column, higher carrier-gas flow rates, and a particle trap) that can hinder the use of mass spectrometric detection - we adopted a single-capillary column configuration. The ability to select different sample loop volumes and adjust the split ratio provides important flexibility for optimizing peak shape, resolution, and S/N performance in GC–MS hydrogen analysis. For higher hydrogen concentrations (≥5000 ppm), a 250 μL sample loop was sufficient to produce well-defined peaks without overloading the detector. In contrast, for lower hydrogen concentrations (<5000 ppm), a larger 2 mL sample loop was necessary to ensure adequate signal intensity. The split ratio was also systematically evaluated and found to produce linear responses within the 5:1 to 50:1 range. Increasing the split ratio improved peak sharpness by reducing band broadening, but excessively high split ratios decreased peak area and sensitivity.

The use of sample loops for gas introduction provides excellent reproducibility in signal response. Figure 2 presents the SIM chromatograms at *m*/*z* = 2 for three replicate injections of a standard gas mixture containing 500 ppm hydrogen, using a 2 mL sample loop. The integrated peak areas from the triplicate runs show very low variability, with a standard deviation corresponding to ±0.56% relative to the mean. This low variability (500 ± 3 ppm) demonstrates the high repeatability of the sampling method and confirms the robustness of the GC–MS-based hydrogen quantification approach.

**Figure 2:**
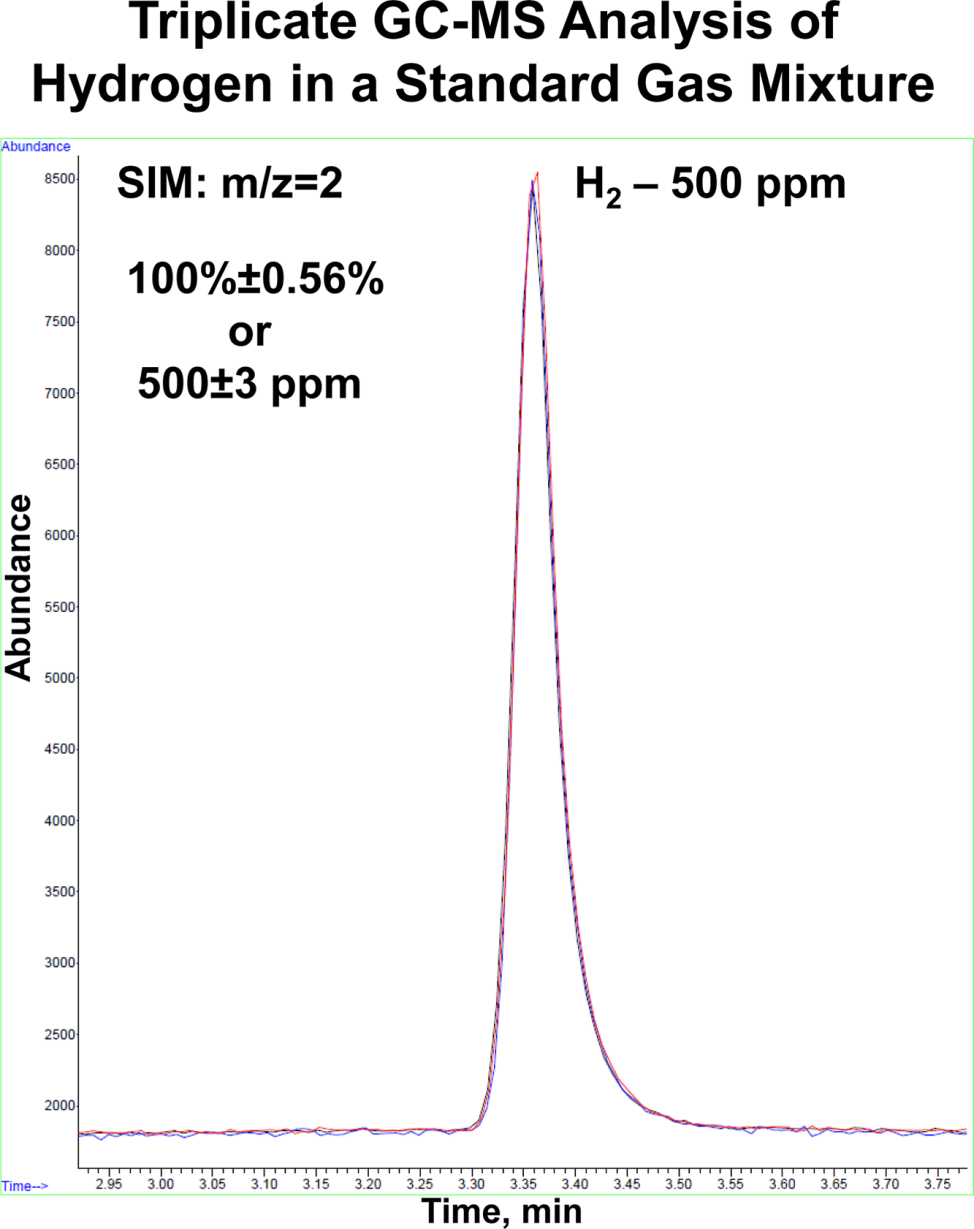
SIM chromatograms at *m*/*z* = 2 for three replicate injections of 500 ppm hydrogen standard gas mixture (N_2_ balance).

Figure 3 shows the calibration curve for hydrogen derived from GC–MS detection with SIM at *m*/*z* = 2 across a concentration range from 5 to 5000 ppm using a 2 mL sample loop with the split ratio of 20:1. The method demonstrates excellent linearity between hydrogen concentration and the corresponding SIM signal intensity with an *R* ^2^ value of 0.9999. Calibration points at 5, 50, 500, and 5000 ppm all fall on the regression line, confirming accurate quantitation across more than 3 orders of magnitude. This strong linear response highlights the robustness and sensitivity of the GC–MS method for hydrogen analysis, enabling reliable quantification from trace levels to percent-level concentrations.

**Figure 3:**
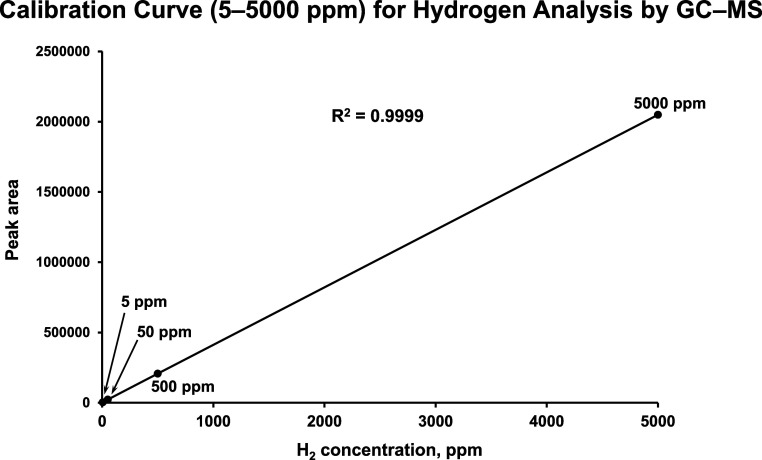
GC–MS calibration curve for hydrogen over 5–5000 ppm concentration range.

Figure 4 presents the gas chromatographic separation and mass spectrometric detection of a refinery gas test mixture using a combined scan (*m*/*z* = 10–160) and SIM (*m*/*z* = 2) acquisition mode. The total ion chromatogram (top panel) demonstrates excellent baseline separation of all major permanent gases and hydrocarbons, including O_2_, N_2_, CO, CH_4_, CO_2_, C_2_H_4_, C_2_H_6_, propane, propylene, *n*-butane, iso-butane, and various C_5_ isomers. Notably, even isobaric species such as N_2_, CO, and C_2_H_4_, each with nominal mass of 28 Da, are fully resolved chromatographically, enabling accurate identification and quantitation despite their identical molecular weights.

**Figure 4:**
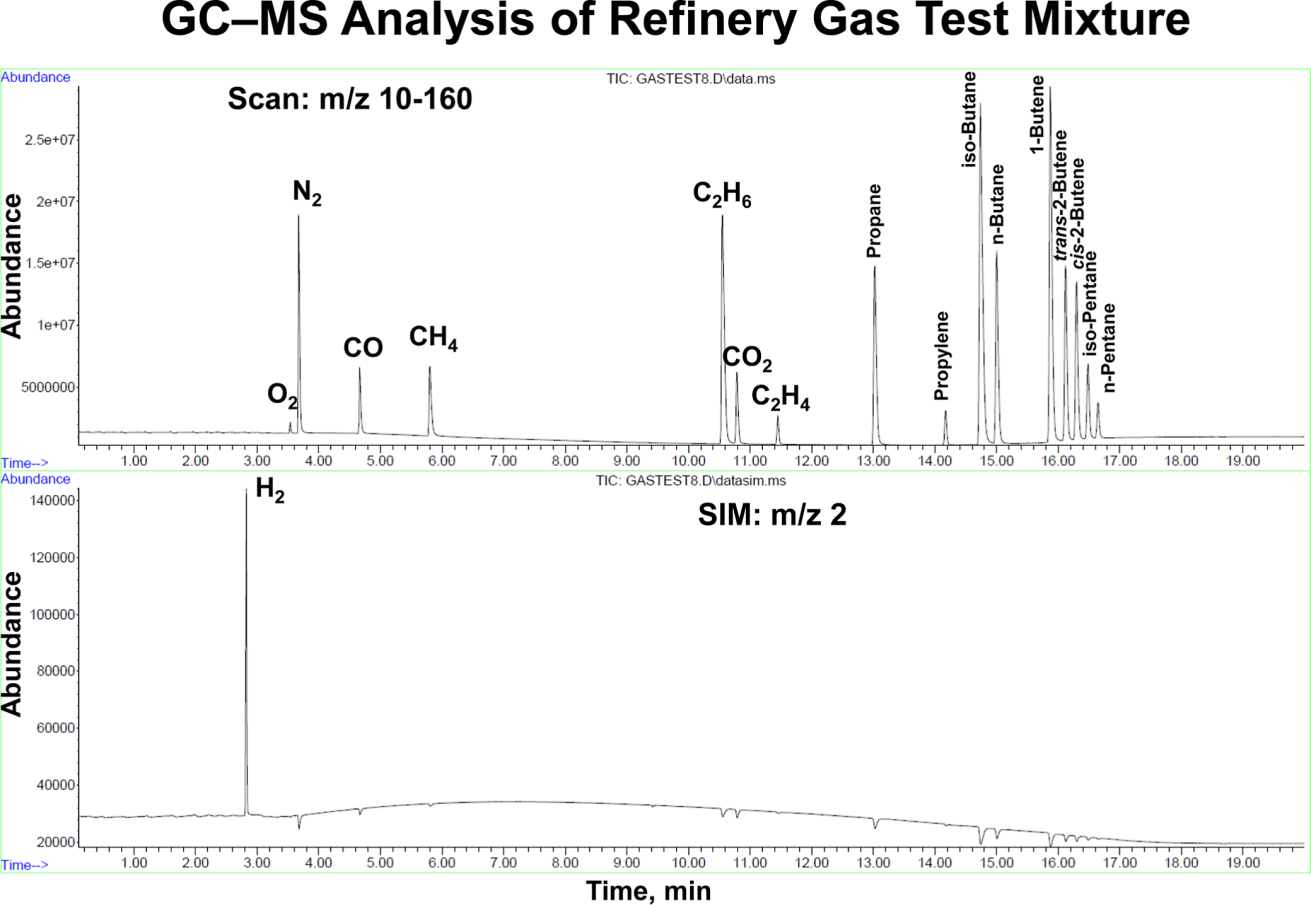
GC–MS analysis of refinery gas mixture. Top. TIC for *m*/*z* = 10–160. Bottom. SIM at *m*/*z* = 2.

The SIM trace at *m*/*z* = 2 (bottom panel) highlights the selective detection of hydrogen. One of the most critical regions for hydrogen separation is between 2 and 7 min, where H_2_ elutes well before O_2_, N_2_, CO, and CH_4_. This complete chromatographic resolution effectively eliminates interference from the quadrupole “zero-blast” effect and suppresses false low-mass signals that typically arise from high concentrations of heavier gases, thereby ensuring reliable and interference-free quantitation of hydrogen. The combination of efficient separation and SIM-based detection provides a robust analytical platform for accurate measurement of hydrogen in complex gas mixtures.

## Conclusions

We successfully developed and validated a robust GC–MS method capable of directly detecting and quantifying hydrogen using EI without the need for dopants, reagent gases, or ion–molecule reaction schemes traditionally required for low-mass. By integrating specialized low-mass tuning procedures, a modified EI source, and a cryogenically cooled single-capillary column, we achieved efficient baseline chromatographic separation of hydrogen from other permanent gases, hydrocarbons, and isobaric species in complex gas mixtures. This separation was essential to eliminate false low-mass signals originating from the quadrupole “zero-blast” effect, and provided accurate, interference-free hydrogen quantitation. The method demonstrated high sensitivity and selectivity across more than 3 orders of magnitude concentration ranges from 5 to 5000 ppm, with excellent linearity (*R* ^2^ = 0.9999). The combination of adjustable sample-loop volumes and tunable split ratios allowed optimization of peak shape and signal-to-noise performance for both trace-level and percent-level hydrogen concentrations. Triplicate analyses showed exceptional reproducibility, with variability below 1%, confirming suitability of the sampling approach for reliable quantitative analysis. Importantly, this approach provides simultaneous compositional information for a wide range of gases in a single analytical run, enabling comprehensive characterization of complex mixtures. This capability is particularly valuable for hydrogen production, storage, fuel-quality monitoring, geologic hydrogen exploration, industrial process control, safety and forensic applications, and environmental assessments where hydrogen must be measured alongside a panel of other gases.

## Notes

This manuscript has been authored by UT-Battelle, LLC, under contract DE-AC05-00OR22725 with the US Department of Energy (DOE). The US government retains and the publisher, by accepting the article for publication, acknowledges that the US government retains a nonexclusive, paid-up, irrevocable, worldwide license to publish or reproduce the published form of this manuscript, or allow others to do so, for US government purposes. DOE will provide public access to these results of federally sponsored research in accordance with the DOE Public Access Plan (https://www.energy.gov/doe-public-access-plan).
